# Thyroid Hormone Mediated Modulation of Energy Expenditure

**DOI:** 10.3390/ijms160716158

**Published:** 2015-07-16

**Authors:** Janina A. Vaitkus, Jared S. Farrar, Francesco S. Celi

**Affiliations:** Division of Endocrinology and Metabolism, Department of Internal Medicine, Virginia Commonwealth University School of Medicine, Richmond, VA 23298, USA; E-Mails: vaitkusj@vcu.edu (J.A.V.); farrarj@vcu.edu (J.S.F.)

**Keywords:** thyroid hormone, mitochondria, uncoupling mechanisms, mitochondrial biogenesis, metabolism, energy expenditure, thyroid hormone receptors

## Abstract

Thyroid hormone (TH) has diverse effects on mitochondria and energy expenditure (EE), generating great interest and research effort into understanding and harnessing these actions for the amelioration and treatment of metabolic disorders, such as obesity and diabetes. Direct effects on ATP utilization are a result of TH’s actions on metabolic cycles and increased cell membrane ion permeability. However, the majority of TH induced EE is thought to be a result of indirect effects, which, in turn, increase capacity for EE. This review discusses the direct actions of TH on EE, and places special emphasis on the indirect actions of TH, which include mitochondrial biogenesis and reduced metabolic efficiency through mitochondrial uncoupling mechanisms. TH analogs and the metabolic actions of T2 are also discussed in the context of targeted modulation of EE. Finally, clinical correlates of TH actions on metabolism are briefly presented.

## 1. Introduction

The maintenance of life is dependent on the metabolism of substrates in the form of carbohydrates, fats, and proteins to provide energy, and in the form of ATP to assure cell integrity and functions. Although in humans the day-to-day variations in energy flux are dramatic, over time, the dynamic equilibrium between energy intake (EI) and energy expenditure (EE) is remarkable. Indeed, a small but sustained imbalance between EE and EI can lead to dramatic and severe clinical presentations, such as obesity or cachexia, both of which represent life-limiting conditions [1,2]. A variety of biochemical pathways are involved in energy metabolism, but in its broadest sense, the common requirement is chemical energy. Basal EE, otherwise defined as resting energy expenditure (REE), is the energy required to maintain basic cell and organ functions. Total EE (TEE) is defined as REE plus the energy consumed during activity (activity EE (AEE)) and diet-induced thermogenesis (DIT), the energy used to metabolize substrates above and beyond the requirements of intestinal tract mobility and absorption [3]. It is important to note that TEE is not static, as REE, AEE, and DIT are all variable and modifiable by a variety of factors. While there are several modulators of REE, and therefore overall EE, the focus of this review will be on thyroid hormone (TH) and its mechanisms of action, particularly on mitochondria. Following the complex integration of various afferent metabolic signals to the hypothalamus [4], TH releasing hormone (TRH) prompts the pituitary gland to secrete thyroid-stimulating hormone (TSH), which in turn activates the thyroid gland to produce and secrete TH [5]. In humans, this is mostly in the form of tetraiodothyronine (also referred to as thyroxine, T4), and to some degree, triiodothyronine (T3) [5]. T4 is then converted into T3 by deiodinase enzymes [5,6], which allow for time- and tissue-specific pre-receptor modulation of the hormonal signal. Most T4 and T3 are bound to thyroxine binding globulin (TBG) and other carriers in circulation, and only unbound or “free” TH exerts biological effects [7]. For the purposes of this review, TH will refer to T3 and T4, while other forms, referred to as TH analogs and “non-classical” THs, will be discussed later.

The critical role of TH in EE modulation has been known for more than a century, starting with the groundbreaking work of Magnus-Levy in 1895 (summarized in [8]). However, each specific mechanism, and in particular their regulatory systems, have yet to be fully elucidated. This review will discuss the developments in knowledge in this area, specifically regarding TH’s role in modulating EE.

## 2. Direct Effects

Direct effects refer to TH actions that inherently cause an increase in ATP utilization. In general, these actions can be further classified into those that are related to metabolic cycles, and those that are related to ion leaks.

### 2.1. Metabolic Cycles

Metabolic cycles, also referred to as substrate or futile cycles, are the combination of two or more reactions which act in a cyclical manner; for a two reaction cycle, the reactions operate in reverse under the control of separate enzymes [9]. In the process of these reactions occurring, ATP is utilized, yet no product is consumed due to the cyclical nature of the products and reactants (hence the designation as a *futile cycle*). Examples of these cycles on the enzymatic level include hexokinase/glucose-6-phosphatase, phosphofructokinase/fructose 1,6-diphosphatase [9], and pyruvate kinase/malic enzyme [10]. Broadly then, futile cycles include such processes as glycolysis/gluconeogenesis, lipolysis (also referred to as fatty acid oxidation)/lipogenesis, and protein turnover, among others [9,11,12]. TH action promotes substrate cycling (reviewed by [9,10,11,13]). Interestingly, Grant and colleagues demonstrated that this increase in cycling results in a reduction in reactive oxygen species (ROS) formation in states of over nutrition [13]. Therefore, TH, by promoting “futile” cycles, plays an important role as an antioxidant in addition to increasing EE. With respect to TEE, however, the EE fraction affected by TH action on metabolic cycles is low compared to other mechanisms discussed later in this review [14,15].

### 2.2. Ion Leaks

**Figure 1:**
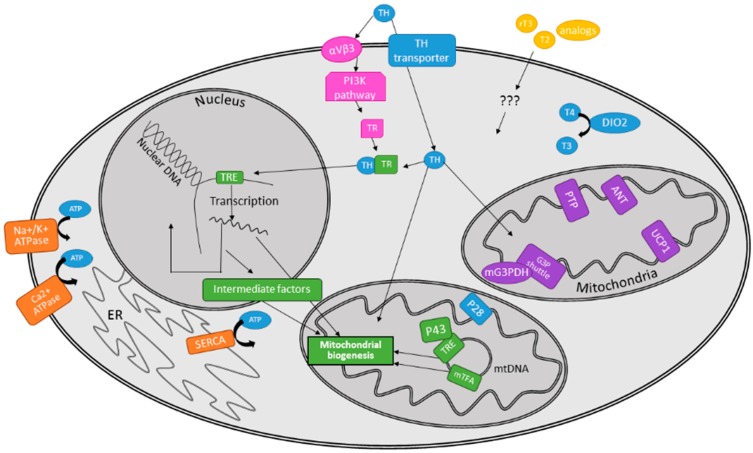
Summary of the mechanisms by which thyroid hormone (TH) modulates energy expenditure (EE) on the cellular level. Orange: Ion leaks. Pink: Non-genomic pathways. Green: Mitochondrial biogenesis resulting from nuclear, intermediate, and mitochondrial-specific pathways. Purple: Uncoupling mechanisms. Yellow: rT3, T2, TH analogs. Blue: TH, ATP, and intermediate steps in TH metabolism and signaling.

A similar yet distinct target of TH activity is an increase in ion leakage, resulting from TH-induced increased cellular membrane permeability to ions. Consequently, a new ion gradient is established, and cells act to re-establish the desired ion concentrations across the membrane of interest at the cost of increased ATP utilization. Two of the most widely studied and understood ion leaks which are induced by TH and lead to futile ion cycling are the Na^+^/K^+^ ATPase and the sarco/endoplasmic reticulum Ca^2+^ ATPase (SERCA) (see Figure 1, orange components). TH action increases both Na^+^ influx and K^+^ efflux into/out of cell plasma membranes, which not only results in increased Na^+^/K^+^ ATPase activity [16], but also increased expression and insertion of these Na^+^/K^+^ ATPases into the plasma membrane [17,18,19,20]. While not as widely discussed, the Ca^2+^ ATPase on the plasma membrane of erythrocytes has also demonstrated regulation and activity modulation by TH [21], supporting the notion that TH exerts non-genomic effects [22] aside from its well-documented transcriptional action (which will be discussed later). TH also mediates leakage of Ca^2+^ from the sarcoplasmic/endoplasmic reticulum (SR/ER) into the cytosol [11], and induces increased expression of ryanodine receptors, which in turn further increase Ca^2+^ efflux out of the SR/ER into the cytosol [23]. Since Ca^2+^ is an extremely important signaling ion and second messenger used by cells, its leakage has the potential to undermine cell survival. In order to restore homeostasis, the cell compensates by increasing Ca^2+^ influx back into the SR/ER via TH-induced expression of SERCA [6,9,24]. Similar to metabolic cycles described above, futile ion cycling has been estimated to play a less substantial role in TH-dependent increases in EE [14,18].

## 3. Indirect Effects

While direct effects have been demonstrated to be important in TH-induced EE, the majority of the thermogenesis induced by TH can be attributed to indirect effects [9]. Indirect effects result in an increased capacity for EE through non-genomic pathways and mitochondrial biogenesis, and also a reduction in metabolic efficiency at the stage of ATP production, by activating uncoupling mechanisms.

### 3.1. Non-Genomic Pathways

TH participates in diverse non-genomic actions which can be initiated at the plasma membrane, in the cytoplasm, or in the mitochondria [7]. These recently discovered non-genomic actions of TH are important for the coordination of normal growth and metabolism, and include regulation of ion channels and oxidative phosphorylation [25]. The principal mediators of non-genomic TH actions on metabolism are the protein kinase signaling cascades [26]. A few examples of non-genomic TH actions are reported below, with comprehensive reviews available elsewhere [6,27]. In an example of plasma membrane TH signaling, T3 binding to the plasma membrane integrin αVβ3 was found to activate the phosphatidylinositol-4,5-bisphosphate 3-kinase (PI3K) pathway, leading to thyroid hormone receptor-α1 (TRα1) receptor shuttling from the cytoplasm to the nucleus (see Figure 1, pink components) and induction of hypoxia-inducible factor 1-α (*HIF1*α) gene expression [28]. Non-genomic TH actions on the cardiovascular system also involve protein-kinase-dependent signaling cascades, which include protein kinase A (PKA), protein kinase C (PKC), PI3K, and mitogen-activated protein kinase (MAPK), with changes in ion channel and pump activities [29]. Other non-genomic actions of TH have been linked to AMP-activated protein kinase (AMPK) and Akt/protein kinase B [30,31,32]. T3 and T2 activate AMPK, a particularly important energy sensor in the cell, resulting in increased fatty acid oxidation, mitochondrial biogenesis, and glucose transporter type 4 (GLUT4) translocation [33,34,35]. Collectively, the non-genomic effects of TH on ion channels and protein kinase signaling cascades may account for a significant component of TH-mediated EE.

### 3.2. Mitochondrial Biogenesis

Of the roughly 1500 mitochondrial genes, the vast majority are housed within the nuclear genome, while the remainder are in the mitochondrial genome [36,37]. In 1992, Wiesner and colleagues demonstrated that the mechanisms of regulation for these two genomes are distinct [38]. TH exerts some of its thermogenic effects by stimulating mitochondrial biogenesis, which has substantial EE implications. Of note, the elevated oxidative capacity due to an increase in the number of mitochondria is not synonymous with an increase in baseline EE, but rather reflects the potential for expansion of respiration in response to an increased demand (such as muscle contraction or adaptive thermogenic response activation) [39].

TH-dependent mitochondrial biogenesis occurs via three mechanisms discussed below: (1) action on nuclear TH receptors; (2) activation of mitochondrial transcription; and (3) expression and activation of intermediate factors that span both the nucleus and the mitochondria (see Figure 1, green components).

#### 3.2.1. Nuclear

In mammals, two genes, *c-ErbA*α and *c-ErbA*β, lead to the production of TH receptors (TRs) (reviewed in [40]). TRα1, TRα2, and TRα3 are the protein products of *c-ErbA*α, yet only the TRα1 isoform binds TH and is functionally relevant [41]. TRβ1 and TRβ2, both of which bind TH, are the products of *c-ErbA*β [42]. TR isoforms are tissue specific, developmentally regulated, and may have distinct functions [43]. All functional TR isoforms contain multiple functional domains, which include a DNA-binding domain (DBD) and a carboxyl-terminal ligand-binding domain (LBD) [7]. The DBD is highly conserved and interacts with specific DNA segments known as TH response elements, or TREs [7]. Thus, TRs are nuclear receptors which modulate gene expression specifically and locally through binding of circulating TH. TRs can exist as monomers, homodimers, and heterodimers; as heterodimers, they can interact with retinoid X receptor (RXR) or retinoic acid receptor (RAR) [44,45]. Through their LBD, TR can also interact with coactivators and corepressors, further modulating TH activity in a tissue specific manner [46]. TH nuclear actions modulate the activities of other transcription factors and coactivators (see Section 3.2.3 below) which are important in metabolic control and the regulation of mitochondrial DNA replication and transcription [47,48,49]. TH also promotes mitochondrial biogenesis through the induction of nuclear encoded mitochondrial genes such as cytochrome c, cytochrome c oxidase subunit IV, and cytochrome c subunit VIIIa [50]. Other TR interacting proteins and TR functions are reviewed extensively elsewhere [6].

#### 3.2.2. Mitochondrial

In addition to the effects described above, TH exerts actions in/on mitochondria [51]. Aside from the nuclear genomic-based pathway of mitochondrial biogenesis, TH also induces mitochondrial genome transcription [25]. TH promotes mitochondrial genome transcription via two distinct mechanisms: directly by binding within the mitochondria to activate transcription machinery, and indirectly by binding to TR nuclear receptors which induce the expression of intermediate factors, which then go on to mitochondria and induce mitochondrial genome-specific gene expression (reviewed by [25] and discussed further in Section 3.2.3 below).

It is important to recognize that direct TH action on mitochondria is not sufficient *per se* to promote mitochondrial biogenesis, since the vast majority of the mitochondrial proteome is encoded by and regulated within the cell’s nuclear genome and cytoplasm [36,37]. Still, there is evidence of direct TH action on the mitochondrial genome. Truncated forms of TRα1, p43 (mitochondrial matrix T3-binding protein) and p28 (inner mitochondrial membrane T3 binding protein), have been isolated in the mitochondrial matrix and inner mitochondrial membrane, respectively [52]. This was a novel and exciting finding, since prior to this discovery there was no knowledge of a non-nuclear TR. Subsequently, Casas and colleagues [53] demonstrated that p43 is indeed restricted to the mitochondria, and that it has similar ligand binding affinity to TRα1, indicating that p43 is the receptor which drives TH mediated transcription of the mitochondrial genome [54,55]. p43 translocates into the mitochondria via fusion to a cytosolic protein [56], and once within the mitochondrial matrix, TH binding to p43 results in p43 interaction with the mitochondrial genome via TREs located in the D loop of the heavy strand [6] to initiate transcription. This mechanism explains the observation of an increased mRNA/rRNA ratio within the mitochondria after exposure to TH [57].

#### 3.2.3. Intermediate Factors

TH also induces mitochondrial biogenesis by bridging nuclear and mitochondrial transcription. This “bridge” is formed by a TH-dependent increase in nuclear expression of a variety of intermediate factors, which can then act on the nucleus, generating a positive feedback loop to either induce nuclear transcription, or to act on the mitochondria to induce mitochondrial transcription [25]. In an extensive review on this topic, Weitzel and Iwen distinguish two distinct classes of intermediate factors: Transcription factors and coactivators [25]. The expression of mitochondrial transcription factor A (mTFA, also referred to as TFAM) is directly regulated by TH, and modulates *in vivo* mitochondrial transcription [58]. Nuclear respiratory factors 1 and 2 (NRF1, NRF2) are transcription factors with multifaceted actions leading to stimulation of mitochondrial biogenesis ([25], and [59] for extensive review). While these intermediate factors function as transcription factors, others function as coactivators of transcription. An example of this class is represented by steroid hormone receptor coactivator 1 (SRC-1), whose action as a coactivator of TH modulates white and brown adipose tissue (BAT) energy balance [60]. Peroxisome proliferator-activated receptor gamma coactivator-1 (PGC-1, both α and β isoforms) are also transcriptionally regulated by TH [25,61] and play a pivotal role in the oxidative capacity of skeletal muscle and BAT (see below). For many metabolism-related genes which are regulated by TH, a putative TRE has yet to be found, further supporting a role for intermediate factors in TH metabolic control [48].

### 3.3. Uncoupling Mechanisms within the Mitochondria

While mitochondrial biogenesis increases the capacity for EE, uncoupling mechanisms manipulate and decrease the efficiency of ATP production within the cell, thereby increasing EE. TH has been demonstrated to play a role in these mechanisms (see Figure 1, purple components), as discussed below.

#### 3.3.1. Uncoupling Proteins

Non-shivering thermogenesis consists of the direct conversion of chemical energy into heat, allowing for a rapid and efficient adaptation to changes in environmental temperature. This ultimately contributed to the evolutionary success of mammals, as it expands the ability to survive in hostile climates [62]. The biochemical hallmark of non-shivering thermogenesis is represented by uncoupling oxidative phosphorylation in the mitochondria, particularly in brown adipose tissue (BAT) [63]. This is accomplished by uncoupling protein-1 (UCP1), which renders the inner membrane of the mitochondria permeable to electrons [64]. This allows for the dissipation of chemical energy as heat, shunting the production of ATP away from the respiration complexes and therefore increasing EE. TH plays an important role in modulating this process. UCP1 transcription is positively regulated by a TRE [65], which therefore implicates TH in this energy-expending activity. Interestingly, in BAT, the intracellular concentration of T3 is relatively independent from the circulating levels of TH, and it is regulated by type 2 deiodinase (DIO2) [66]. DIO2 is driven by the β-adrenergic cyclic AMP (cAMP) signaling cascade [67], which promotes an increase in intracellular conversion of the prohormone T4 into T3, the ligand for the TH receptor. This signal pathway ultimately assures a time- and tissue-specific modulation of TH action relatively independent of circulating TH levels [66], with obvious effects on EE [68].

In addition to UCP1, which is the hallmark of brown adipose tissue transcriptome signature, other structurally-related proteins with putative uncoupling properties have been described in other tissues. UCP2 and UCP3 are the most well studied and their transcription is induced by TH [69,70]. UCP3, which is predominantly expressed in skeletal muscle, has been associated with TH-induced modulation of REE [71] and fatty acid peroxide-induced mitochondrial uncoupling [72]. Additional actions of uncoupling proteins are reviewed elsewhere [9,73].

#### 3.3.2. PCG-1α

While TH action directly stimulates EE in the mitochondria by promoting the uncoupling of substrate oxidation from ADP phosphorylation, TH also augments the overall capacity for non-shivering thermogenesis and therefore EE by positively regulating the transcription of PGC-1α, the master regulator of brown and “beige” adipocyte differentiation and mitochondria proliferation [74]. PGC-1α is also an important modulator of EE in muscle, where it promotes the switch from glycolytic function toward oxidative metabolism [75]. Interestingly, PGC-1α also plays a role in modulating the relative ratio between the transcriptionally active isoform of the TH receptor (TRα1) and the “inactive” TRα2 isoform devoid of the ligand binding domain, thereby generating a sort of intracellular negative feedback [76].

#### 3.3.3. Mitochondrial Permeability Transition Pore

Mitochondrial uncoupling by T3 is driven by gating of the mitochondrial permeability transition pore (PTP) [77]. Previous studies have shown that mitochondrial PTP opening is exquisitely sensitive to mitochondrial Ca^2+^ [78], which is classically increased in states of cell stress [79]. Prolonged opening of the PTP results in mitochondrial depolarization and swelling, and if PTP conductance is sufficiently elevated, mitochondrial rupture will ensue with release of pro-apoptotic proteins and programmed cell death [80]. Interestingly, in addition to its historic role in apoptosis, recent evidence has emerged to implicate PTP in TH-mediated EE. Yehuda-Shnaidman *et al*. found that mitochondrial uncoupling by T3 required activation of the endoplasmic reticulum inositol 1,4,5-triphosphate receptor 1 (IP(3)R1), suggesting an upstream role for IP(3)R1 in the action of T3 on EE [77]. This study indicated a novel target for TH-dependent mitochondrial EE and the potential for targeting future TH analogs to this pathway. While much research is still necessary in this area, it is possible that IP(3)R1 may result in increased PTP opening, uncoupling, and therefore EE. For a more extensive discussion of the mitochondrial PTP and its role in TH induced EE, please see a recent review by Yehuda-Shnaidman and colleagues [9].

#### 3.3.4. ANT

The mitochondrial adenosine diphosphate/adenosine triphosphate (ADP/ATP) translocase, or ANT, forms a gated pore in the inner mitochondrial membrane, allowing ADP to flow into the mitochondrial matrix and ATP in the opposite direction towards the cytoplasm [81]. ANT serves an important role in oxidative phosphorylation by controlling the flow of ADP substrate into the mitochondria, which is subsequently phosphorylated to ATP. As an important regulator of mitochondrial EE, ANT and cytosolic and mitochondrial ADP/ATP ratios were an early focus of studies into TH stimulated EE [82,83]. Indeed, in 1985, Seitz and colleagues demonstrated that T3 could rapidly increase mitochondrial respiration, ATP regeneration, and the activity of ANT in rat liver [82]. T3 stimulation of ANT was later confirmed and more expansively studied in rat liver mitochondrial isolates [84]. Mowbray and colleagues proposed a model in which T3 caused covalent modification of ANT, promoting a conformation with elevated ADP and cation flux [85]. This study directly linked T3 to mitochondrial uncoupling and provided evidence for the role of TH in shunting substrate towards heat generation in the mitochondria instead of ATP production. Brand *et al.* later demonstrated that basal proton conductance in the mitochondria of mice lacking ANT1 was half that of wild-type controls; firmly establishing the role of ANT in mitochondrial basal uncoupling [86] and therefore EE. Finally, ANT may serve an important role in long-term adaptive thermogenesis. In their study, Ukropec *et al*. found that mice lacking UCP1 were able to induce ANT1/2 and other proteins to compensate for long-term cold exposure [87]. Taken together, these data suggest an important role for ANT in the uncoupling of mitochondrial respiration.

#### 3.3.5. Glycerol-3-Phosphate Shuttle

In order for the electron transport chain to produce ATP, reducing equivalents must also be present in the inner mitochondrial matrix, in addition to ADP as described above. Two mechanisms that allow for this are the malate-aspartate shuttle and the glycerol-3-phosphate (G3P) shuttle [11]. These shuttles differ in the resultant nucleotides which they provide to the electron transport chain within the mitochondria; the malate-aspartate shuttle provides NADH, while the G3P shuttle provides FADH_2_ [9]. This seemingly minute difference has substantial implications with respect to energy balance, as subsequent oxidative phosphorylation of NADH results in the synthesis of 3 ATP, compared with only 2 ATP for a FADH_2_ molecule (reviewed in [9]). In this sense, the G3P shuttle is less metabolically efficient, and therefore, if its action is upregulated, it can function as an energy dissipation mechanism. Indeed, TH regulates the G3P shuttle at the level of FADH-dependent mitochondrial glycerol-3-phosphate dehydrogenase (mG3PDH) [9]. mG3PDH is located on the outer side of the mitochondrial inner membrane and allows for the conversion of G3P into dihydroxyacetone phosphate (DHAP) [88]. In this conversion, FADH_2_ is formed and shuttled into complex II of the electron transport chain. Silva and colleagues studied a transgenic *mG3PDH*−/− mouse model and found significantly higher levels of TH ([89], and reviewed in [11]). This evidence suggests a clear role for TH in thermogenesis created by the G3P shuttle. However, total oxygen consumption was not reduced as drastically as expected (only a 7%–10% reduction in the transgenic *mG3PDH*−/− mouse compared to controls) [89]. This suggests that compensatory mechanisms exist to lessen the reduction in EE when mG3PDH is not present. 

## 4. TH Analogs and Non-Classical THs

### 4.1. TH Analogs

The diverse effects of TH on metabolism prompted researchers to study its use as a potential therapeutic for obesity and dyslipidemia. However, supra-physiologic TH levels cause a toxic state, and their systemic effects such as tachycardia, bone loss, muscle wasting, and neuropsychiatric disturbances prevent therapeutic use [90]. For these reasons, supplementing TH in euthyroid individuals for the treatment of obesity was abandoned. A logical development from research on TH actions has been the isolation and synthesis of TH derivatives with favorable side effect profiles, or “ideal” target-tissue distribution, to exploit beneficial metabolic effects while minimizing toxicity and systemic adverse effects. Newer TH derivatives have been developed with tissue and TRβ specificity (reviewed in [91,92]) (see Figure 1, yellow components). By focusing on TRβ selectivity, the adverse cardiac effects of TH have been reduced due to the low expression of TRβ receptors in the heart [93]. Tissue specificity has focused on the actions of TH in the liver, in part because synthetic TH derivatives could be made with high first-pass metabolism in the liver and greatly lowered serum concentrations [92]. The synthetic TH analog GC-1 (sobetirome) has been shown to prevent or reduce hepatosteatosis in a rat model [94] and can reduce serum triglyceride and cholesterol levels without significant side-effects on heart rate [95]. Additionally, GC-1 has been shown to increase EE and prevent fat accumulation in female rats [96].

### 4.2. Non-Classical THs

In addition to the “classic” THs T4 and T3, other naturally occurring “non-classical” THs may have physiological actions or be exploited therapeutically in the modulation of EE (see Figure 1, yellow components). The mechanisms of action of non-classical THs, which include 3,3′,5′-triiodothyronine (rT3), thyronamines (TAMs), and 3,5-diiodothyronine (T2) have been recently reviewed in detail elsewhere [8,97,98,99]. In this review, we will briefly discuss the metabolic actions of T2. T2 is found at picomolar serum concentrations in humans [100], and at similar concentrations, T2 is able to stimulate oxygen consumption in the isolated perfused livers of hypothyroid rats [101]. T2 has also been shown to directly and rapidly stimulate mitochondrial activity [102] and elevate resting EE in rats [103]. Subsequently, it was demonstrated that T2 can prevent high fat diet-induced hepatosteatosis and obesity in rats by stimulating mitochondrial uncoupling and decreasing ATP synthesis [104,105]. Furthermore, T2 can treat obesity and hepatosteatosis [106] and prevent high fat diet-induced insulin resistance in rats [107]. Finally, recent experimental evidence indicates that T2 is able to activate BAT-dependent thermogenesis and enhance mitochondrial respiration in hypothyroid rats [108]. In an attempt towards translating experimental findings to humans, Antonelli *et al*. administered T2 to healthy, euthyroid subjects and monitored changes in body weight, resting metabolic rate (RMR) and thyroid function [109]. Compared to baseline, T2-treated subjects had a significant elevation in RMR, reduced body weight, and normal thyroid and cardiac function, while no changes in any of these metrics were observed in the placebo group. Within the limitation of a very small proof-of-concept trial, this study further supports the potential of T2 to therapeutically increase RMR and reduce body weight.

## 5. Clinical Correlates

The recent discovery of naturally occurring mutations in the *TR*α gene [110] has provided the opportunity to assess *in vivo* the differential effects of TH signaling by comparing and contrasting the effects of TH receptor α and β mutations on energy metabolism. The human phenotype of resistance to TH (RTH) secondary to mutations in the *TR*β gene is commonly characterized by a combination of hyper- and hypothyroid hormonal signaling at different end-organ tissues, with an overall increase in EE [111]. Conversely, the recently described syndrome of RTH secondary to *TR*α mutations is characterized by increased adiposity and decreased EE [112], in keeping with the predominance of *TR*α in high energy demanding tissues such as myocardium. Interestingly, while both isoforms are present in BAT [113], *TR*β is the prevalent isoform, playing a critical role in the adaptive thermogenic response [114]. The data therefore strongly suggest that the modulatory activity of lipolysis and EE by *TRα* is primarily due to indirect effects, rather than direct action on the mitochondria. Interestingly, an association between polymorphisms in the *TR*α locus and increased body mass index has been reported, supporting the role of this isoform in energy metabolism [115]. From a clinical standpoint, these findings suggest that the development of a receptor isoform or tissue-specific TH agonist may represent a viable strategy to modulate end-organ targets or pathways with precision, without generating undesirable side effects.

## 6. Conclusions and Final Remarks

TH has pleiotropic effects on mitochondria and energy expenditure. The modulation of TH’s actions is critical in the delivery of time and tissue specific signaling. The effects of TH in increasing energy expenditure via modulation of the adaptive thermogenesis response, coupled with the ability of increasing respiratory capacity by regulating mitochondrial biogenesis, are augmented by the increase in TH’s non-mitochondrial effects on futile cycles and ion transport. Finally, the opportunity to selectively modulate TH effects represents a promising therapeutic target for the amelioration of a wide range of metabolic disorders.
